# Enzymatic Synthesis of a Novel Pterostilbene α-Glucoside by the Combination of Cyclodextrin Glucanotransferase and Amyloglucosidase

**DOI:** 10.3390/molecules23061271

**Published:** 2018-05-25

**Authors:** José L. González-Alfonso, David Rodrigo-Frutos, Efres Belmonte-Reche, Pablo Peñalver, Ana Poveda, Jesús Jiménez-Barbero, Antonio O. Ballesteros, Yoshihiko Hirose, Julio Polaina, Juan C. Morales, María Fernández-Lobato, Francisco J. Plou

**Affiliations:** 1 Instituto de Catálisis y Petroleoquímica, CSIC, 28049 Madrid, Spain; josel.g@csic.es (J.L.G.-A.); a.ballesteros@icp.csic.es (A.O.B.); 2 Centro de Biología Molecular Severo Ochoa (CSIC-UAM), Departamento Biología Molecular, UAM, 28049 Madrid, Spain; drodrigo@cbm.csic.es (D.R.-F.); mfernandez@cbm.csic.es (M.F.-L.); 3 Instituto de Parasitología y Biomedicina “Lopez-Neyra”, CSIC, PTS Granada, 18016 Armilla, Granada, Spain; efresbr@ipb.csic.es (E.B.-R.); pablo@ipb.csic.es (P.P.); jcmorales@ipb.csic.es (J.C.M.); 4 Center for Cooperative Research in Biosciences, Parque Científico Tecnológico de Bizkaia, 48160 Derio, Biscay, Spain; apoveda@cicbiogune.es (A.P.); jjbarbero@cicbiogune.es (J.J.-B.); 5 Enzyme Techno, Naganatsu, Ogaki, Gifu 503-0997, Japan; yohirose@octn.jp; 6 Instituto de Agroquímica y Tecnología de Alimentos, CSIC, 46980 Valencia, Spain; jpolaina@iata.csic.es

**Keywords:** polyphenols, glycosylation, stilbenes, pterostilbene, cyclodextrin glycosyltransferase, enzymatic glucosylation, amyloglucosidase

## Abstract

The synthesis of a novel α-glucosylated derivative of pterostilbene was performed by a transglycosylation reaction using starch as glucosyl donor, catalyzed by cyclodextrin glucanotransferase (CGTase) from *Thermoanaerobacter* sp. The reaction was carried out in a buffer containing 20% (*v*/*v*) DMSO to enhance the solubility of pterostilbene. Due to the formation of several polyglucosylated products with CGTase, the yield of monoglucoside was increased by the treatment with a recombinant amyloglucosidase (STA1) from *Saccharomyces cerevisiae (*var. *diastaticus)*. This enzyme was not able to hydrolyze the linkage between the glucose and pterostilbene. The monoglucoside was isolated and characterized by combining ESI-MS and 2D-NMR methods. Pterostilbene α-d-glucopyranoside is a novel compound. The α-glucosylation of pterostilbene enhanced its solubility in water to approximately 0.1 g/L. The α-glucosylation caused a slight loss of antioxidant activity towards ABTS˙^+^ radicals. Pterostilbene α-d-glucopyranoside was less toxic than pterostilbene for human SH-S5Y5 neurons, MRC5 fibroblasts and HT-29 colon cancer cells, and similar for RAW 264.7 macrophages.

## 1. Introduction

Dietary phytophenols exhibit a wide range of bioactivities such as antioxidant, antihypertensive, antitumoral, bactericidal, neuroprotective and anti-inflammatory [1]. Pterostilbene (*trans*-3,5-dimethoxy-4′-hydroxystilbene) is a natural phytoalexin mainly found in blueberries and *Pterocarpus marsupium* heartwood; chemically, it is a dimethyl ether analog of resveratrol. However, the pharmacological activities (in vitro and in vivo) of pterostilbene are usually better than those of resveratrol [2], as the methoxyl groups at positions 3- and 5- enhance membrane permeability and metabolic stability [3]. It is well reported that pterostilbene possesses excellent pharmacological benefits for the prevention and treatment of various types of cancer [4,5], Alzheimer’s disease [6], vascular dementia [7], obesity [8] or diabetes [9]. In addition, it exerts beneficial anti-aging effects by lowering several processes such as oxidative damage, inflammation, telomere attrition and cell senescence [10].

The notable hydrophobicity of many phytophenols, including resveratrol and pterostilbene, seems to be related with their poor absorption in vivo [11]. In this context, various polyphenols are glycosylated in nature and this glycosylation could play a major role in their absorption [12]. There are some evidences that the sugar moiety of polyphenols can modulate the bioavailability [13], bioactivity [14], solubility [15] and partition coefficient [16,17] of these molecules. Glycosylation may also protect polyphenols from oxygen and/or light degradation and boost their efficiency to prevent skin photo-ageing damages [18].

For glycosylation of phytophenols, the use of enzymes is very attractive due to their excellent specificity, increased availability and to the mild reaction conditions (moderate temperature, atmospheric pressure, pH close to neutrality, etc.) [19,20,21]. The synthesis of glycosidic bonds can be catalyzed by glycosyltransferases (EC 2.4), which are classified into three main mechanistic groups: (1) Leloir-type glycosyltransferases, which require sugar nucleotides; (2) non-Leloir glycosyltransferases, which employ sugar-1-phosphates; and (3) transglycosidases, which use simple and widely available carbohydrates such as sucrose, lactose or starch [21,22]. In addition, glycosidases (glycoside hydrolases, EC 3.2) can also be utilized for in vitro formation of glycosidic bonds under appropriate reaction conditions [23,24].

In this work, we describe the enzymatic synthesis of an alpha-glucosyl derivative of pterostilbene by a transglycosylation reaction catalyzed by a transglycosidase, namely cyclodextrin glucanotransferase or glycosyltransferase (CGTase, EC 2.4.1.19) from *Thermoanaerobacter* sp., which employs starch as glucosyl donor [25]. This enzyme was previously employed by our group in the α–glucosylation of resveratrol [26]. To increase the yield of monoglucoside, we added a hydrolytic step catalyzed by a recombinant amyloglucosidase from *Saccharomyces cerevisiae (*var. *diastaticus).* The antioxidant properties and toxicity of the synthesized compound was further assessed.

## 2. Results and Discussion

### 2.1. Enzymatic Glucosylation of Pterostilbene

Several glycosidases and glycosyltransferases were screened for the glycosidation of pterostilbene (Table 1). The reaction medium contained 20% (*v*/*v*) DMSO to favor the solubilization of the antioxidant, which is almost insoluble in water. Among the enzymes tested, only cyclodextrin glycosyltransferase (CGTase) gave rise to a significant formation of glycosylated products. The enzyme from *Thermoanaerobacter* sp. (Toruzyme 3.0L) worked better than that from *Bacillus macerans* (CGTase Amano). The transglycosylation activity of CGTase is well reported, as it is able to glucosylate other phenolic compounds such as resveratrol [26,27], catechin [28], hydroquinone [29], kaempferol [30] or genistein [31].

The reactions were carried out in absence of light to avoid the photoisomerization of *trans*-pterostilbene to the *cis*-isomer [32]. This isomerization changes the overall configuration and decreases the biological activity [5]. We incubated the reaction products in deuterated water at room temperature in presence of light and observed by NMR that the isomerization *trans-cis* was quite fast.

**Figure 1:**
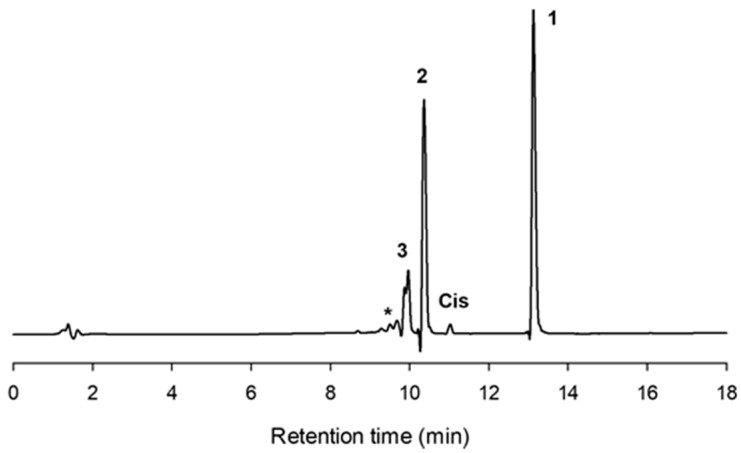
HPLC chromatogram (C-18 column) showing the reaction mixture after 10 h with the CGTase from *Thermoanaerobacter* sp. Peaks: (1) pterostilbene; (2) pterostilbene monoglucoside; (3) pterostilbene diglucoside; (Cis) *cis*-pterostilbene; (*) polyglucosylated derivatives. Reaction conditions: pterostilbene (5 mg/mL), starch (100 mg/mL), CGTase from *Thermoanaerobacter* sp. (10% *v*/*v*), 20% DMSO (*v*/*v*), 60 °C, 150 rpm.

Figure 1 illustrates the chromatogram using a C-18 column of a typical mixture obtained with CGTase from *Thermoanaerobacter* sp. after 10 h reaction. As shown, the formation of *cis*-pterostilbene was nearly negligible. Two major peaks (2 and 3) with higher polarity than pterostilbene appeared on the left of the chromatogram, which should correspond to glycosylated derivatives.

Peaks 2 and 3 were purified by semipreparative HPLC on a C-18 column as described. The molecular weight was determined by MS using electrospray and QTOF analyzer (see Supplementary Material, Figures S1 and S2). For compound 2, the major peak in the mass spectrum in positive mode was at *m*/*z* 441.15 corresponding to the M + [Na]^+^ ion of the pterostilbene monoglucoside. For compound 3, the main signal in the mass spectrum in negative mode was at *m*/*z* 579.21 belonging to the M − [H]^+^ ion of the pterostilbene diglucoside.

The other peaks in the chromatogram of Figure 1 (marked with an asterisk) probably corresponded to derivatives with a higher degree of polymerization (triglucoside, tetraglucoside, etc.). It is well reported that CGTase forms homologous series of glucosylated products via coupling or disproportionation reactions [33,34].

**Figure 2:**
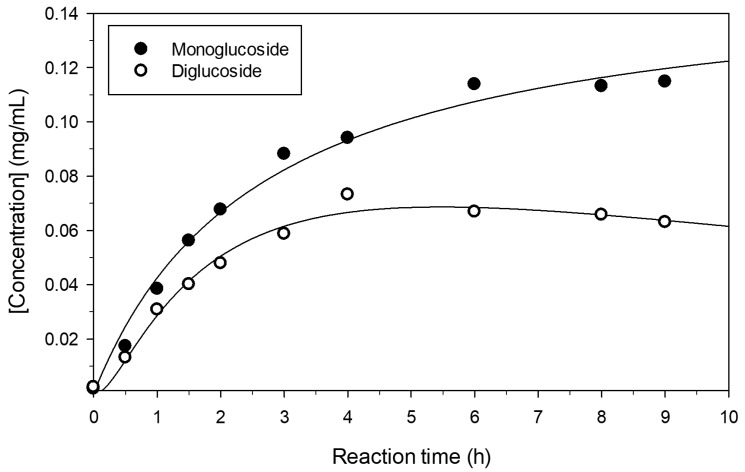
Progress of the formation of the main pterostilbene glucosides. Reaction conditions were as described in Figure 1.

The progress of pterostilbene glucosylation under the assayed conditions was further studied (Figure 2). After 10 h, the concentration of products varied only slightly. The maximum concentrations of mono- and di-glucoside were approximately 0.12 and 0.06 mg/mL, respectively.

### 2.2. Characterization of the Monoglucosylated Derivative

The structure of the glucosylated derivative was deduced by using standard NMR (see Table 2 and Figure S3 of the Supplementary Material). The analysis of the NMR data showed that glycosidation of the pterostilbene ring took place at the free phenolic position, as deduced from the downfield shift of the proton signals belonging to this aromatic ring with respect to the free pterostilbene moiety. The α-configuration of the glycosidic linkage was stablished by the value of the ^3^*J* coupling between the protons H1 and H2 of the glucose moiety, which is 3.6 Hz. ^1^H- and ^13^C-NMR chemical shifts δ for position 1- of Glc also supported this configuration (δ_H_ = 5.41 and δ_C_ = 97.62 ppm, respectively). The β-glucosylated derivative has been described [35], and for that case the values for the same parameters are: ^3^*J*_H1H2_ = 8Hz, δ_H_ = 4,87 ppm, δ_C_ = 105 ppm. The NMR spectroscopic data for pterostilbene 4′-*O*-α-d-glucopyranoside is summarized in Table 1.

**Figure 3:**
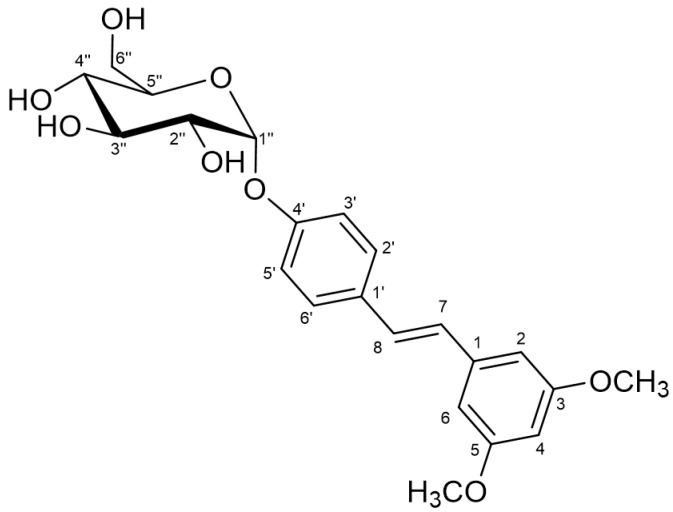
Chemical structure of pterostilbene 4′-*O*-α-d-glucopyranoside.

On the basis of the above results, the proposed non-ambiguous structure for the isolated compound was pteroestilbene 4′-*O*-α-d-glucopyranoside (Figure 3). To our knowledge, this is a novel compound. The HRMS of the glucoside is presented in Supplementary Material (Figure S4). Previous studies on glucosylation of pterostilbene led to the formation of β-glucosides. Thus, the group of Hamada employed a recombinant glucosyltransferase from *Phytolacca americana* (or the cultured cells) to synthesize the 4′-*O*-β-d-glucoside using UDP-glucose as donor [36,37]. Although the authors reported 95% yield, the initial concentration of pterostilbene was extremely low (50 µM). The same product was obtained by a chemical process that implies the use of NaOH, solvent mixtures of CHCl_3_/H_2_O and tetrabutylammonium bromide as phase transfer catalyst [38].

### 2.3. Increase of Monoglucoside Yield by Treatment with Amyloglucosidase

Once the 4′-*O*-α-d-glucoside was synthesized, CGTase was able to further glucosylate this molecule thus giving the so-called homologous series, formed by a mixture of mono-, di-, tri-, tetra- and even higher glucosides. In previous studies on CGTase-catalyzed transglycosylations, it is reported that the bonds between glucosyl groups are majorly α(1→4) [33,39,40]. Although the CGTase displays a low hydrolytic activity on such linkages [41], we incorporated a glycosidase to accelerate the hydrolytic process and thus increase the yield of monoglucoside. A similar strategy was successfully employed for the glucosylation of inositol [42], genistein [31], ascorbic acid [43] or hydroquinone [29]. In the case of pterostilbene, after 10 h reaction with CGTase, we incubated the mixture with a recombinant glucoamylase from *S. cerevisiae* (var. *diastaticus*) [44] expressed in *Pichia pastoris* (unpublished data). The purity of these amylolytic enzymes is critical for the control and reproducibility of the hydrolytic step.

**Figure 4:**
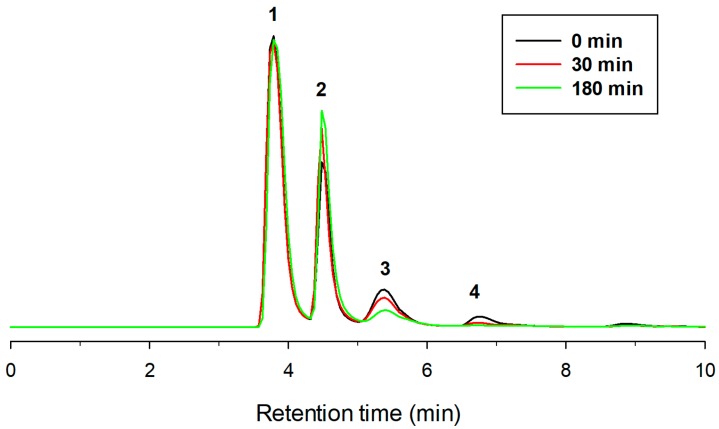
HPLC chromatograms (NH2 column) showing the post-treatment with amyloglucosidase during 3 h of the reaction mixture obtained with CGTase (10 h, standard conditions). Peaks: (1) *trans*-pterostilbene; (2) pterostilbene monoglucoside; (3) pterostilbene diglucoside; (4) pterostilbene triglucoside.

**Figure 5:**
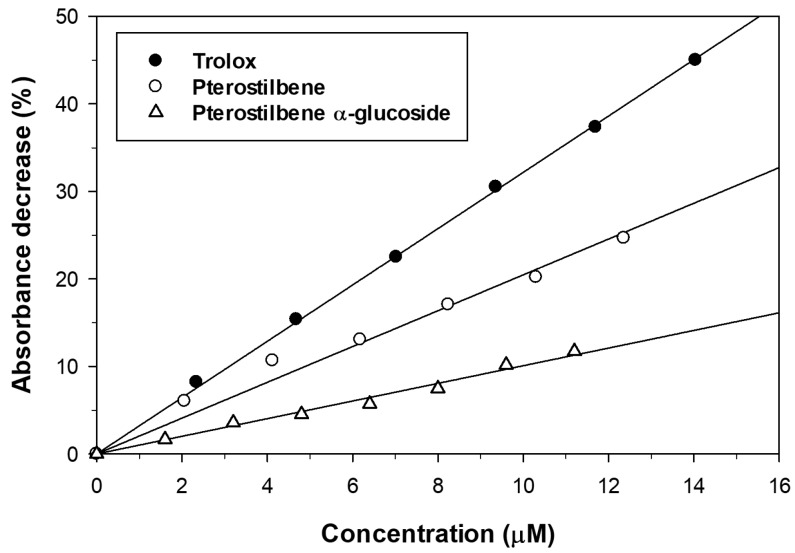
Effect of pterostilbene and its α-glucoside on ABTS˙^+^ reduction. Trolox was used as antioxidant reference compound.

As illustrated in Figure 4, the HPLC analysis using a NH2 column showed that, after 3 h incubation with amyloglucosidase, di- and triglucoside peaks (3 and 4) mostly disappeared, with a concomitant increase of the monoglucoside derivative. The chromatogram of Figure 5 also indicated that a percentage of diglucoside was not hydrolyzed by amyloglucosidase, which implies that other bonds, apart from α(1→4), could be present in minor amounts. It is worth emphasizing that the α-linkage between glucose and pterostilbene is resistant to the action of amyloglucosidase. This resistance was also reported in the case of ascorbic acid [43] and hydroquinone [29], but not with genistein α-glucoside [31].

After the treatment with amyloglucosidase, the maximum concentration of monoglucosylated pterostilbene increased from 0.11 mg/mL to 0.15 mg/mL. This concentration is one order of magnitude higher than that reported by Uesugi et al. in the synthesis of the corresponding 4′-β-glucoside by a glucosyltransferase from *P. americana* (in soluble form or employing the cultured cells) [37].

### 2.4. Effect of Glucosylation on Solubility and Antioxidant Activity of Pterostilbene

The aqueous solubility of pterostilbene 4′-*O*-α-d-glucopyranoside at 30 °C was compared with that of the aglycon. Under such conditions, pterostilbene was almost insoluble whilst the α-d-glucoside showed a solubility of 98 ± 2 mg/L. This is a new example in which glycosylation increases the solubility of plant polyphenols [15,45].

We also studied the antioxidant activity of pterostilbene and its α-glucosylated derivative by the Trolox Equivalent Antioxidant Capacity (TEAC) assay. The ABTS˙^+^ radical cation was generated from ABTS with potassium persulfate and the addition of the antioxidant reduced it to ABTS thus decreasing the absorbance. The antioxidants were assayed at different concentrations and the decrease of absorbance of ABTS˙^+^ solution was monitored at 734 nm during 6 min. The results are represented in Figure 5. The incorporation of an α-glucosyl moiety to the pterostilbene caused a significant loss of scavenging activity towards ABTS˙^+^ radicals. A similar decrease of antioxidant activity was described for the β-glucoside [37]. The TEAC values, calculated from the slopes of linear regressions represented in Figure 5, are summarized in Table 3. Both the aglycon and the monoglucosylated derivative displayed TEAC values lower than that obtained for Trolox.

### 2.5. Effect of Glucosylation on Toxicity of Pterostilbene

Pterostilbene has sparked a lot of interest as a potential therapeutic agent in the prevention or treatment of cancer, diabetes, obesity or neurodegenerative diseases [46]. Its bioactivity is closely related with its antioxidant, radical scavenging, anti-apoptotic and anti-inflammatory properties [47]. Several studies confirmed the potential of pterostilbene to promote healthy ageing [10]. As a novel compound, there are no reports on the bioactivity and pharmacokinetic properties of 4′-α-d-glucoside of pterostilbene, or its potential as prodrug. However, the 4′-β-glucoside was reported to display promising biological properties, for example its antiallergic activity (i.e., histamine release inhibitory activity) was higher than that of the aglycon [48]. This compound was also able to induce collagen expression [49]. Shimoda et al. demonstrated that the inhibition activity of pterostilbene towards phosphodiesterase (PDE) was enhanced by β-glucosylation [36]. PDE inhibitors possess a notable interest as neuroprotectors and to minimize the deleterious effects of aging.

**Figure 6:**
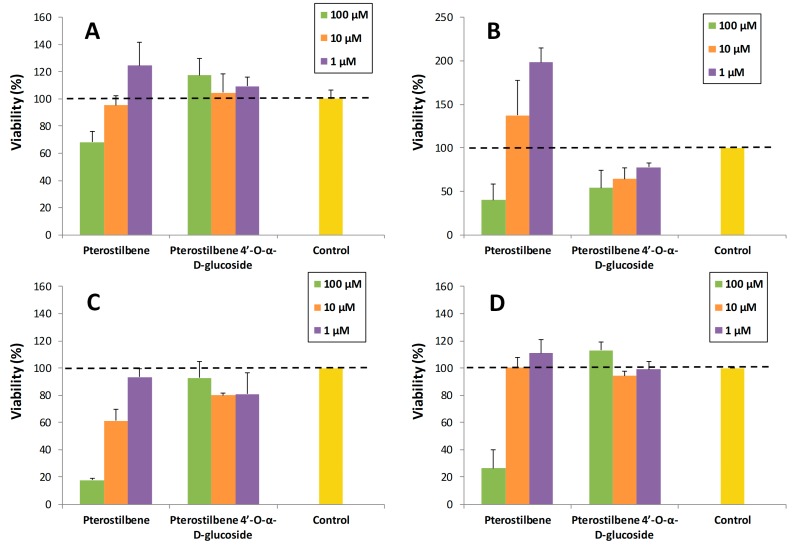
Cell viability assays of pterostilbene and its 4′-α-glucoside on: (**A**) SH-SY5Y neuronal cells; (**B**) RAW 264.7 macrophages; (**C**) MRC5 fibroblasts; (**D**) HT-29 colon cancer cells. The values are referred to the control (cells containing 1% DMSO). The data is expressed as mean ± SD (*n* = 8).

A preliminary study was carried out to explore the potential of pterostilbene 4′-*O*-α-d-glucopyranoside. In particular, its toxicity on various cell lines (SH-SY5Y neuronal cells, RAW 264.7 macrophages, MRC5 fibroblasts and HT-29 colon cancer cells) was compared with that of pterostilbene. For this purpose, the viability of cells in the presence of the compounds at three concentrations (1, 10 and 100 µM) was determined (Figure 6). The final DMSO percentage in each cell was adjusted to 1% (*v*/*v).* The values were referred to the control (cells containing 1% DMSO). As shown, the α-glucoside was significantly less toxic than the aglycon for neurons, fibroblasts and colon cancer cells (Figure 6A,C,D), especially at high concentration (100 µM). Regarding macrophages, the pterostilbene was less toxic than the glucosylated derivative (Figure 6B).

With the exception of macrophages, this trend has been previously observed for similar glucosylated phenolic compounds such as piceid (resveratrol 3-*O*-β-d-glucopyranoside). Our group [50] and others [51,52,53] have observed less cytotoxicity for piceid than for resveratrol in very different cell lines such as human embryonic kidney cell line (HEK-293), human keratinocytes (HaCaT), human breast adenocarcinoma cell line (MDA-MB231) or liver hepatocellular carcinoma (HepG2). As it has been proposed by Su et al. [53], the reason that piceid showed lower cytotoxicity than resveratrol at the same concentration was probably due to the fact that the uptake of the piceid by cells was less efficient than that of resveratrol. A similar reasoning may apply for our case on the comparison between pterostilbene 4′-*O*-α-d-glucopyranoside and its aglycone.

## 3. Materials and Methods

### 3.1. Chemicals

Partially hydrolyzed starch from potato (Paselli SA2) was from Avebe (Foxhol, The Netherlands). ABTS [2,2′-azino-bis(3-ethylbenzothiazoline-6-sulphonic acid)] and (*R*)-Trolox (6-hydroxy-2,5,7,8-tetramethylchroman-2-carboxylic acid) were purchased from Sigma-Aldrich (Madrid, Spain). All other reagents and solvents were of the highest available purity and used as purchased.

### 3.2. Enzymes

The β-fructofuranosidase from *Saccharomyces cerevisiae* and cyclodextrin glucanotransferase (CGTase) from *Thermoanaerobacter* sp. (Toruzyme 3.0L) were kindly provided by Novozymes A/S (Bagsværd, Denmark). Toruzyme 3.0L was partially purified by a PD-10 desalting column (GE Healthcare, Madrid, Spain). CGTase from *Bacillus macerans* (cyclodextrin glucanotransferase) and α-glucosidase from *Aspergillus niger* (Transglucosidase L) were kindly supplied by Amano Enzyme Inc. (Oxfordshire, UK). The β-galactosidase from *Bacillus circulans* (Biolactase NTL Conc. 2x) was provided from Biocon (Barcelona, Spain). The β-fructofuranosidase from *Xanthophyllomyces dendrorhous* was obtained as previously described [54].

For the production of recombinant amyloglucosidase from *S. cerevisiae (*var. *diastaticus)* (STA1), a 2.35 kb *Eco*RI/*Sac*II DNA fragment containing the *STA1* gene from *S. cerevisiae (*var. *diastaticus)* [55] was cloned in the *Pichia pastoris* expression vector pPICZalpha. The resulting plasmid was used to transform *P. pastoris* strain X-33 according to the manual for protein expression in *Pichia* (Invitrogen, Carlsbad, CA, USA). To obtain STA1 protein expressed in *P. pastoris*, transformants were grown, and the heterologous protein was produced and purified as described in a previous work [54]. Briefly, extracellular fraction of yeast cells showing maximun amyloglucosidase activity (500 mL; 6 days of culture; 1.2 U/mL) was concentrated (approx. 20 mL; 20 U/mL) through 100,000 MWCO PES membranes by using a Vivaflow 50 system (Sartorius, Goettingen, Germany) and applied to a DEAE-Sephacel chromatography column (3 mL) equilibrated with 20 mL Tris-HCl (pH 7.0). Active fractions (1 mL) eluted at 0.1 M NaCl were pooled (10 mL; 8 U/mL) and concentrated using Amicon Ultracel 100 K (Merck Millipore Ltd., Cork, Ireland). Purity of the protein was confirmed by SDS-PAGE (8% polyacrylamide) using standard methodology. The hydrolytic activity of STA1 was measured using the dinitrosalicylic acid method adapted to a 96-well microplate scale. The reaction mixture (50 µL) contained 20 g/L soluble starch (Sigma-Aldrich, St. Louis, USA) in 0.1 M sodium citrate pH 5.0, the enzyme solution (5 µL) was added and incubated at 37 °C for 10 min. Glucose (0.2–3.0 g/L) was used for the calibration curve. One unit of hydrolytic activity was defined as that corresponding to the release of 1 μmol of reducing sugar per min.

### 3.3. Enzymatic Glucosylation of Pterostilbene

Pterostilbene (5 mg) and partially hydrolyzed starch (100 mg) were dissolved in a mixture of H_2_O (0.7 mL) and DMSO (0.2 mL). Partially purified CGTase from *Thermoanaerobacter* sp. (0.1 mL) was then added to a final concentration of 10% (*v*/*v*). The mixture was kept at 60 °C in an orbital shaker (model SI50, Stuart Scientific, Staffordshire, UK) at 150 rpm. Aliquots (150 μL) were withdrawn at intervals, passed through 0.45 μm nylon filters (Cosela), diluted with 150 μL acetonitrile to stop the reaction and analyzed by HPLC. For the hydrolysis of polyglucosylated derivatives, the reaction with CGTase was first carried out under the above conditions during 10 h. Then, it was cooled and centrifuged 15 min at 7800× *g* to remove non-solubilized starch. The supernatant was collected and amyloglucosidase from *S. diastaticus* (STA1) (40 µL, final activity 3.6 U/mL) was then added and the mixture kept at 40 °C. Aliquots of 150 µL were taken during 3 h, diluted with 150 µL of acetonitrile and analyzed by HPLC.

### 3.4. High-Performance Liquid Chromatography (HPLC)

HPLC analysis was performed using a quaternary pump (model 600, Waters, Milford, MA, USA) coupled to an autosampler (model ProStar 420, Varian Inc., Palo Alto, CA, USA). The column was a Kromasil-NH2 (4.6 mm × 250 mm, 5 μm, Analisis Vinicos, Tomelloso, Spain). The injection volume was 20 µL. The temperature of the column was kept constant at 40 °C. The detection of peaks was carried out using a photodiode array detector (ProStar, Varian) and peaks were analyzed using the Varian Star LC workstation 6.41. The mobile phase was acetonitrile/water 80:20 (*v*/*v)* at 0.8 mL/min during 10 min. Both solvents contained 0.1% (*v*/*v*) of formic acid. The analysis was also performed with a Zorbax Eclipse Plus C18 column (4.6 mm × 100 mm, 3.5 μm, Agilent Technologies, Santa Clara, CA, USA). The starting mobile phase was H_2_O/acetonitrile 85:15 (*v*/*v*), and a gradient to H_2_O/acetonitrile 5:95 (*v*/*v)* was performed in 12 min. This mobile phase was maintained for 2 min and the column was further equilibrated to the initial conditions. The flow rate was 0.8 mL/min, and the column temperature was kept constant at 40 °C.

### 3.5. Purification of Pterostilbene Glucosides

The glucosylation of pterostilbene was scaled up. The reaction mixture contained pterostilbene (50.7 mg), soluble starch (1 g), partially purified Toruzyme 3.0 L (1 mL), 7 mL of water and 2 mL of DMSO. The mixture was incubated for 16 h at 60 °C with orbital shaking (model SI50, Stuart Scientific) at 150 rpm. The mixture was then cooled and concentrated, and the glucosylated derivatives of pterostilbene were purified using semipreparative HPLC. The column was a Zorbax Eclipse XDBC-18 (9.4 mm × 250 mm, 5 μm, Agilent Technologies). A three-way flow splitter at 1/10 (Accurate, LC Packings) was employed. The starting mobile phase was H_2_O/acetonitrile 85:15 (*v*/*v*), and a gradient to H_2_O/acetonitrile 5:95 (*v*/*v*) was performed in 12 min. Both solvents contained 0.1% (*v*/*v*) of formic acid. The flow rate was 7 mL/min, and the column temperature was kept constant at 40 °C. After collecting the glucosylated products, the mobile phase was eliminated by rotary evaporation in an R-210 rotavapor (Büchi, Flawil, Switzerland). The isolated products were characterized by mass spectroscopy and NMR.

### 3.6. Mass Spectrometry (MS)

The molecular weight of purified pterostilbene derivatives was assessed using a mass spectrometer with hybrid QTOF analyzer (model QSTAR, Pulsar i, AB Sciex, Framingham, MA, USA). Samples were analyzed by direct infusion and ionized by electrospray (with methanol containing 1% of NH_4_OH as ionizing phase) in negative reflector mode.

### 3.7. Nuclear Magnetic Resonance (NMR)

The structure of the monoglucosylated derivative was determined using a combination of 1D and 2D (COSY, DEPT-HSQC, NOESY) standard NMR techniques. The spectra of the sample, dissolved in DMSO-*d*_6_ (*ca.* 7 mM), were recorded on a IVDr 600 spectrometer (Bruker, Billerica, MA, USA) equipped with a BBI probe with gradients in the Z axis, at a temperature of 300 K. Chemical shifts were expressed in parts per million (ppm). Residual DMSO-d5 signal was used as internal reference (2.5 ppm). All the employed pulse sequences were provided by Bruker. For the DEPT-HSQC experiment, values of 8 ppm and 1 K points, for the 1H dimension, and 165 ppm and 256 points for the 13C dimension, were used. For the homonuclear COSY and NOESY experiments, 8 ppm windows were used with a 1 K × 256 point matrix. For the NOESY the mixing time was 500 ms.

### 3.8. Trolox Equivalent Antioxidant Capacity (TEAC) Assay

The ABTS˙^+^ cation was generated from ABTS solution (7 mM) with potassium persulfate (2.45 mM) for 15 h. The radical cation absorbed at 734 nm and was stable for two days. ABTS˙^+^ was diluted in ethanol to 0.7 ± 0.02 absorbance units at 734 nm. The antioxidant solution (20 µL, between 20 and 210 µM) was added to 230 µL of adjusted ABTS˙^+^ solution. The decrease of absorbance of ABTS˙^+^ solution was monitored at 734 nm during 6 min using a microplate reader (model Versamax, Molecular Devices, San Jose, CA, USA). The decrease of absorbance was determined measuring the area under the curve. The reference antioxidant was (*R*)-Trolox. The TEAC values were expressed as the concentration (µM) at which the compound decreases the same absorbance as 1 µM (*R*)-Trolox.

### 3.9. Solubility Assay

A saturated solution of pterostilbene or its monoglucoside was prepared in water at 30 °C with stirring for 24 h. Then, the solution was filtered, conveniently diluted with water and analyzed by HPLC as previously described.

### 3.10. Toxicity Tests

SH-S5Y5 neurons were cultured in collagen-pretreated petri-dishes with DMEM-F12 medium supplemented with penicillin/streptomycin and 10% inactivated fetal bovine serum (iFBS). RAW 264.7 macrophages and HT-29 colon cancer cells were cultured in DMEM high glucose medium supplemented with penicillin/streptomycin and 10% iFBS. MRC5 were cultured in DMEM low glucose medium supplemented with glutamine, penicillin/streptomycin and 10% iFBS.

Neuron assays were done in collagen-pretreated 96 well plates by seeding 2 × 10^4^ neurons per well in a 100 µL volume and with 24 h of incubation before the compound addition. Macrophage assays were done in 96 well plates by seeding 2.5 × 10^4^ macrophages per well in a 100 µL volume with 4 h of incubation before the compound addition. MRC5 and HT-29 assays were done in 96 well plates by seeding 5 × 10^4^ cells per well in a 100 µL volume and with 24 h of incubation before the compound addition. Tested compounds dissolved in DMSO were then added at different final concentrations (1, 10 and 100 µM) to determine compound toxicity. Final DMSO percentage in each cell was adjusted to 1%. Cell viability was evaluated during 24 h (SH-SY5Y and RAW 264.7 cells) or 48 h (MRC5 and HT-29 cells) after the addition of the compounds, following the mitochondrial MTT assay, according to manufacturer. Averages and standard deviations of at least eight different readings from various experiments were calculated.

## 4. Conclusions

We have synthesized a novel α-glucoside of pterostilbene under mild conditions (H_2_O:DMSO 80:20 as solvent, 60 °C) using partially hydrolyzed starch as glucosyl donor and CGTase as biocatalyst. Although the antioxidant activity decreased upon glucosylation, the monoglucoside presented higher solubility in water than the aglycon, and it was also less toxic for several cell lines. The synthesized derivative could be of interest in the nutraceutical, cosmetic and biomedical industries, as occurs with other glucosides of polyphenols obtained by biocatalytic routes [56]. However, further studies on its in vivo bioavailability and biological activity are required to determine its full potential.

## Figures and Tables

**Table 1:** Screened enzymes for the glycosylation of pterostilbene.

Enzyme	Source	Glycosyl Donor	[Monoglucoside] (mg/mL) ^a^
β-galactosidase	*Bacillus circulans*	Lactose	0.0015
α-glucosidase	*Aspergillus niger*	Maltose	0.0002
β-fructofuranosidase	*Saccharomyces cerevisiae*	Sucrose	−
	*Xanthophyllomyces dendrorhous*	Sucrose	−
CGTase	*Thermoanaerobacter* sp.	Starch	0.1148
	*Bacillus macerans*	Starch	0.0129

^a^ Reaction conditions: pterostilbene (5 mg/mL), glycosyl donor (100 mg/mL), enzyme (10% *v*/*v*), 20% DMSO (*v*/*v*), 60 °C, 150 rpm, 20 h.

**Table 2:** NMR Spectroscopic Data (600 MHz, DMSO-*d*_6_) for pterostilbene 4′-*O*-α-d-glucopyranoside.

Position	δ_C,_ Type ^a^	δ_H_ (*J* in Hz)
2/6	103.98, CH	6.75 (d, *J* = 2.2 Hz, 2H)
4	99.28, CH	6.40 (t, *J* = 2.2 Hz, 1H)
OMe (3/5)	54.90, CH_3_	3.78 (s, 6H)
7	128.15, CH	7.22 (d, *J* = 16.3 Hz, 1H)
8	126.40, CH	7.05 (d, *J* = 16.3 Hz, 1H)
2′/6′	127.39, CH	7.53 (d, *J* = 8.7 Hz, 2H)
3′/5′	116.85, CH	7.10 (d, *J* = 8.7 Hz, 2H)
1′′	97.62, CH	5.41 (d, *J* = 3.6 Hz, 1H)
2′′	71.29, CH	3.38 (ddd, *J* = 9.6, 6.3, 3.6 Hz, 1H)
3′′	72.84, CH	3.63 (td, *J* = 9.6, 5.0 Hz, 1H)
4′′	69.65, CH	3.20 (td, *J* = 9.6, 5.8 Hz, 1H)
5′′	73.49, CH	3.44–3.51 (m, 1H)^b^
6′′	60.45, CH_2_	3.44–3.51 (m, 1H)^b^/3.57 (dd, *J* = 10.0, 5.6 Hz, 1H)
OH2′′	-	5.05 (d, *J* = 6.3 Hz, 1H)
OH3′′	-	4.93 (d, *J* = 5.0 Hz, 1H)
OH4′′	-	4.97 (d, *J* = 5.8 Hz, 1H)
OH6′′	-	4.47 (t, *J* = 5.7 Hz, 1H)

^a^ Obtained from HSQC-edited spectrum; ^b^ Overlapped signals in ^1^H.

**Table 3:** TEAC values of pterostilbene and its α-glucoside.

Compound	Slope	R^2^	TEAC
Trolox	3.22	0.996	1.00
Pterostilbene	1.74	0.990	2.40
Pterostilbene 4′-*O*-α-d-glucoside	1.03	0.980	4.05

## Supplementary Materials

The following figures are available online. Figure S1: ESI-MS spectrum (positive mode) of the isolated pterostilbene monoglucoside, Figure S2: ESI-MS spectrum (negative mode) of the isolated pterostilbene diglucoside. Figure S3: NMR Spectra (600 MHz, DMSO-d6, 298 K) for pteroestilbene 4′-*O*-α-d-glucopyranoside: (a) 1H spectrum; (b) COSY; (c) HSQC- edited; (d) NOESY 500 ms. Figure S4: HRMS and elementary composition calculation for pterostilbene 4′-*O*-α-d-glucopyranoside.
