# Supplementary material for: Enzymatic Synthesis of a Novel Pterostilbene α-Glucoside by the Combination of Cyclodextrin Glucanotransferase and Amyloglucosidase
Source: Molecules. 2018 May 25;23(6):1271. doi: 10.3390/molecules23061271 (PMC6100302; doi:10.3390/molecules23061271)
Supplement: Supplementary file 1 [file molecules-23-01271-s001.pdf]

Enzymatic synthesis of a novel pterostilbene α-glucoside by the combination of cyclodextrin glucanotransferase and amyloglucosidase

Jose L. Gonzalez-Alfonso[1], David Rodrigo-Frutos[2], Efres Belmonte-Reche[3], Pablo Peñalver[3], Ana Poveda[4], Jesús Jimenez-Barbero[4], Antonio O. Ballesteros[1], Yoshihiko Hirose[5], Julio Polaina[6], Juan C. Morales[3], María Fernández-Lobato[2] and Francisco J. Plou[1,*]

1 Instituto de Catálisis y Petroleoquímica, CSIC, 28049 Madrid, Spain; josel.g@csic.es (J.L.G.-A.); a.ballesteros@icp.csic.es (A.O.B.)
2 Centro de Biología Molecular Severo Ochoa (CSIC-UAM), Departamento Biología Molecular, UAM, 28049 Madrid, Spain.; mfernandez@cbm.csic.es (M.F.-L.), drodrigo@cbm.csic.es (D.R.-F.)
3 Instituto de Parasitología y Biomedicina "Lopez-Neyra", CSIC, PTS Granada, 18016 Armilla,, Granada, Spain; efresbr@ipb.csic.es (E.B.-R.); pablo@ipb.csic.es (P.P.); jcmorales@ipb.csic.es (J.C.M.)
4 Center for Cooperative Research in Biosciences, Parque Científico Tecnológico de Bizkaia, 48160 Derio, Biscay, Spain; apoveda@cicbiogune.es (A.P); jjbarbero@cicbiogune.es (J.J.-B)
5 Enzyme Techno, Naganatsu, Ogaki, Gifu 503-0997, Japan; yohirose@octn.jp
6 Instituto de Agroquímica y Tecnología de Alimentos, CSIC, Valencia, Spain; jpolaina@iata.csic.es

* Correspondence: fplou@icp.csic.es; Tel.: +34-91-585-4869

Figure S1: ESI-MS spectrum (positive mode) of the isolated pterostilbene monoglucoside.

Figure S2: ESI-MS spectrum (negative mode) of the isolated pterostilbene diglucoside.

Figure S3: NMR Spectra (600 MHz, DMSO-d6, 298 K) for pteroestilbene 4′-O-α-D-glucopyranoside: (a) ^1H spectrum; (b) COSY; (c) HSQC- edited; (d) NOESY 500 ms.

Figure S4: HRMS and elementary composition calculation for pterostilbene 4′-O-α-D-glucopyranoside.

Figure S1.

ESI-MS spectrum (positive mode) of the isolated pterostilbene monoglucoside.

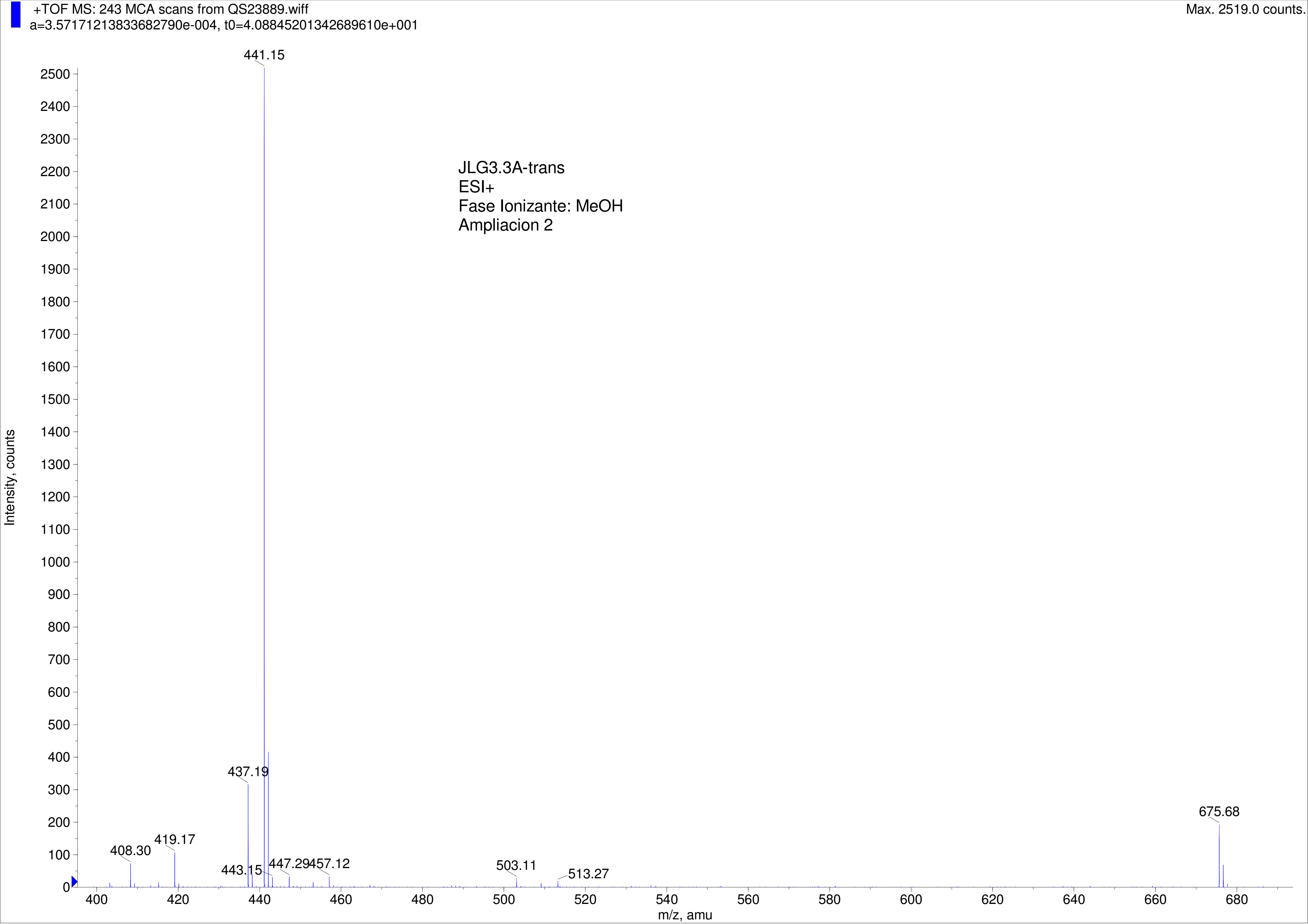
+TOF MS: 243 MCA scans from QS23889.wiff
a=3.57171213833682790e-004, t0=4.08845201342689610e+001
Max. 2519.0 counts.
JLG3.3A-trans
ESI+
Fase Ionizante: MeOH
Ampliacion 2
441.15
437.19
408.30
419.17
443.15
447.29
457.12
503.11
513.27
675.68
Intensity, counts
m/z, amu

Figure S2.

ESI-MS spectrum (negative mode) of the isolated pterostilbene diglucoside.

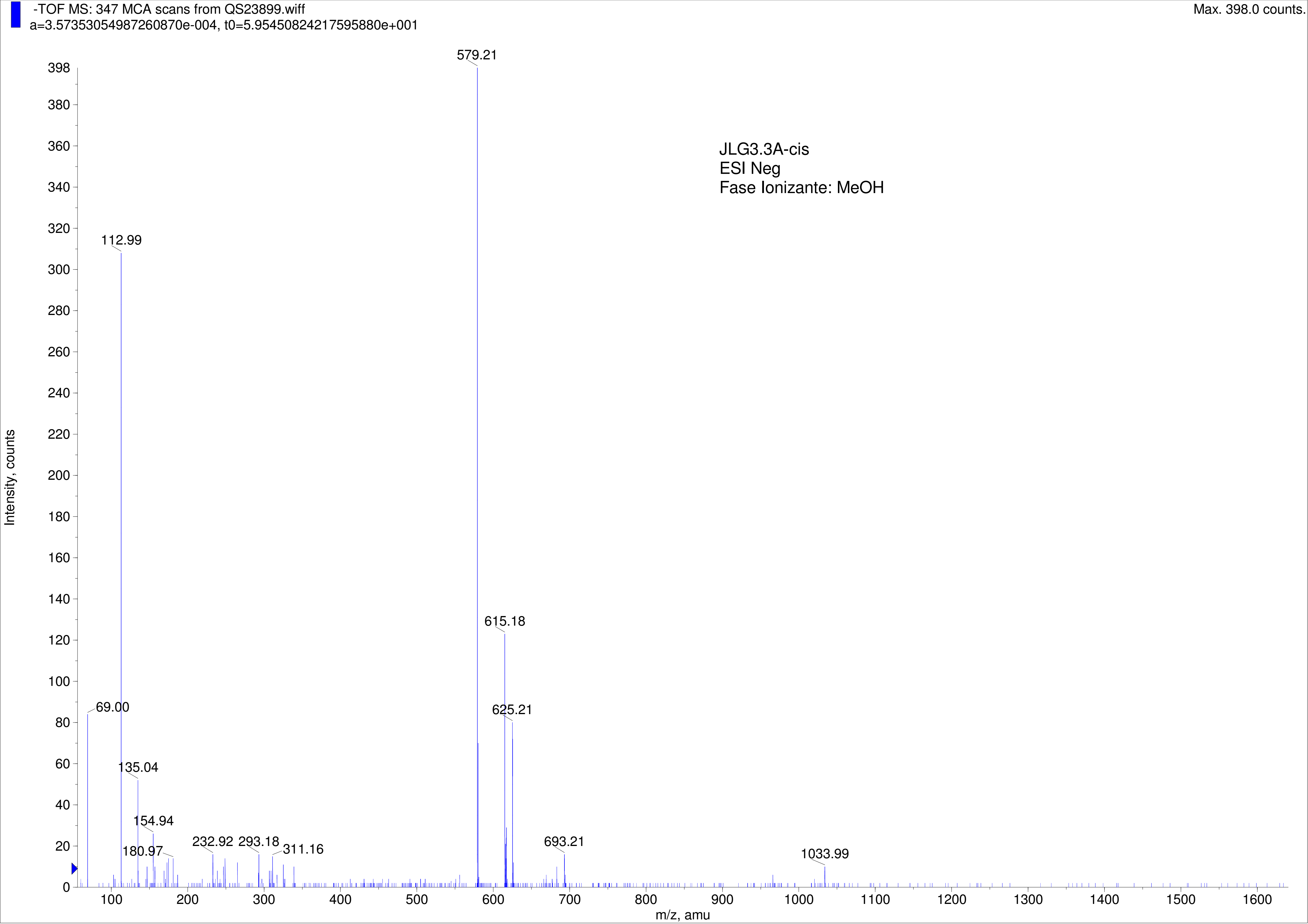
-TOF MS: 347 MCA scans from QS23899.wiff
a=3.57353054987260870e-004, t0=5.95450824217595880e+001
Max. 398.0 counts.
JLG3.3A-cis
ESI Neg
Fase Ionizante: MeOH
579.21
112.99
615.18
625.21
69.00
135.04
154.94
180.97
232.92
293.18
311.16
693.21
1033.99
Intensity, counts
m/z, amu

Figure S3.

NMR Spectra (600 MHz, DMSO-d6, 298 K) for pteroestilbene 4′-O-α-D-glucopyranoside: (a) ^{1}H spectrum; (b) COSY; (c) HSQC- edited; (d) NOESY 500 ms.

NMR Spectra (600 MHz, DMSO-d_6, 298K) for pteroestilbene 4'-O-α-D-glucopyranoside

(a) ^{1}H spectrum

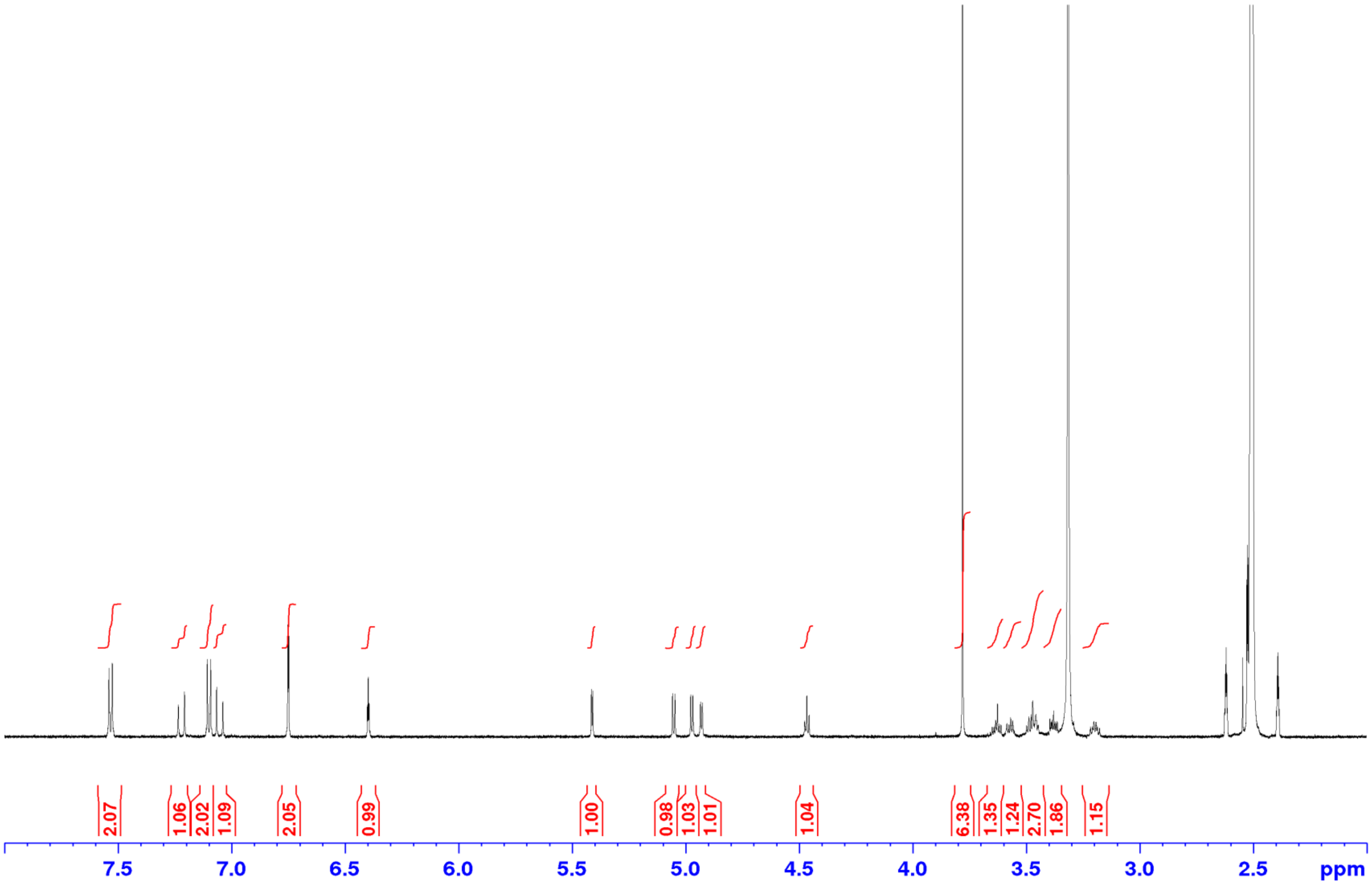

(b) COSY

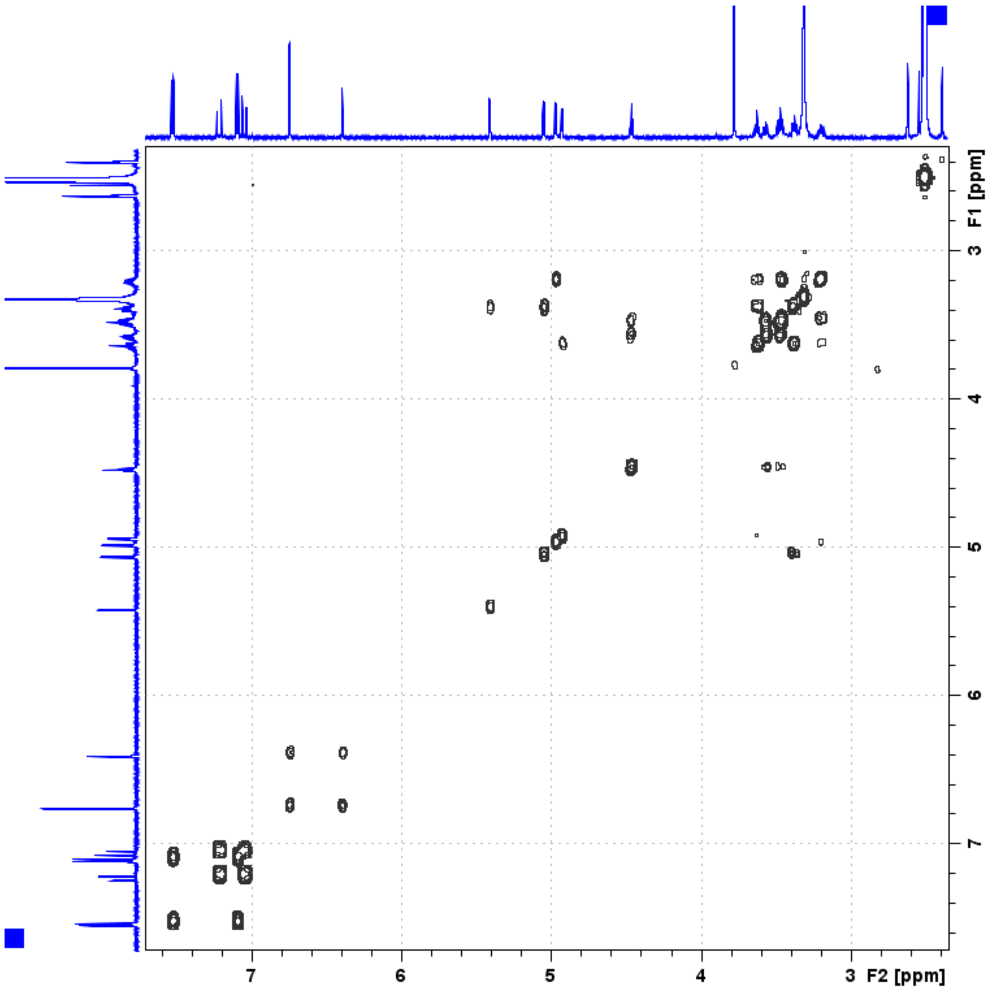

(c) HSQC- edited

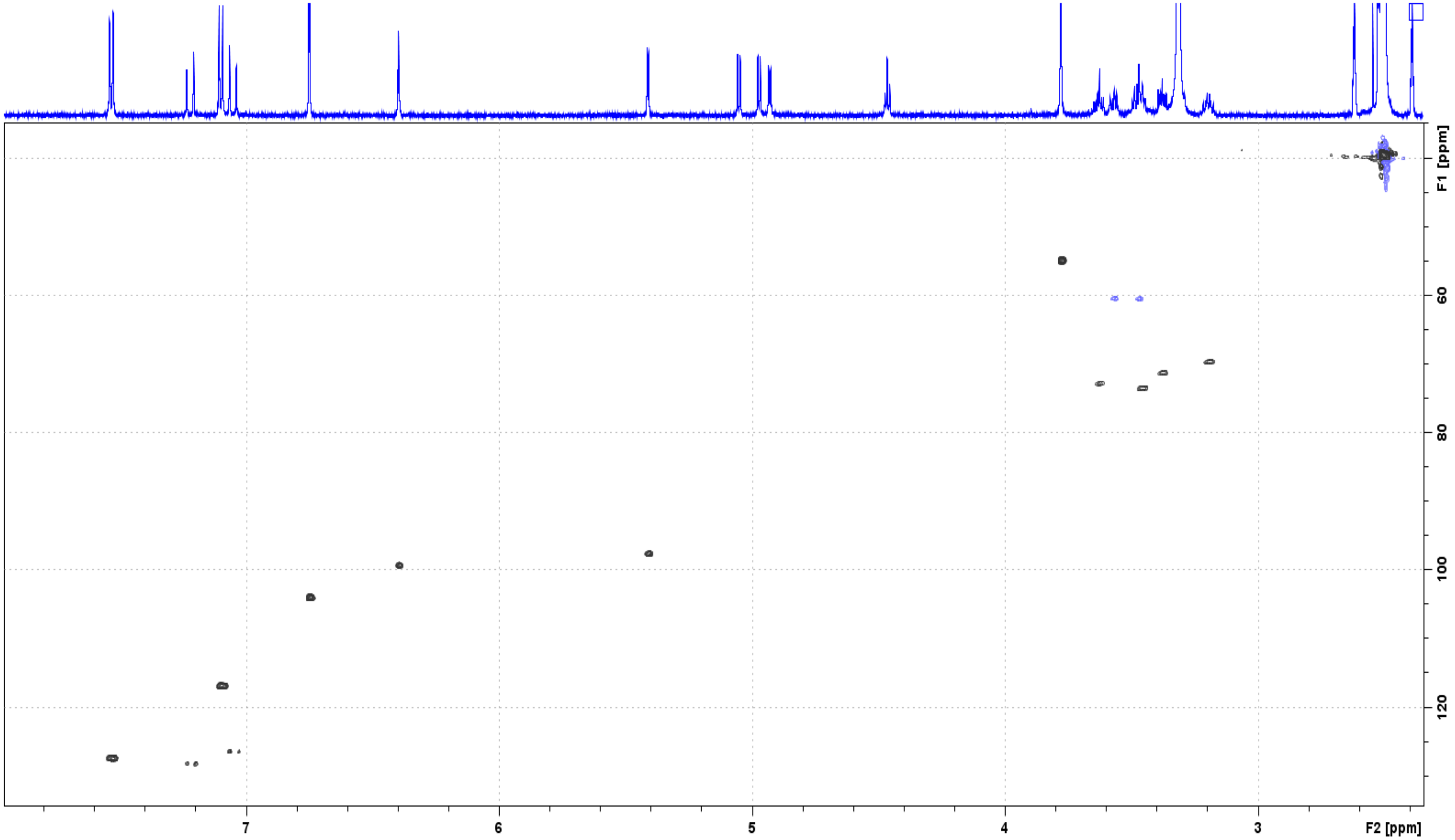

(d) NOESY 500ms

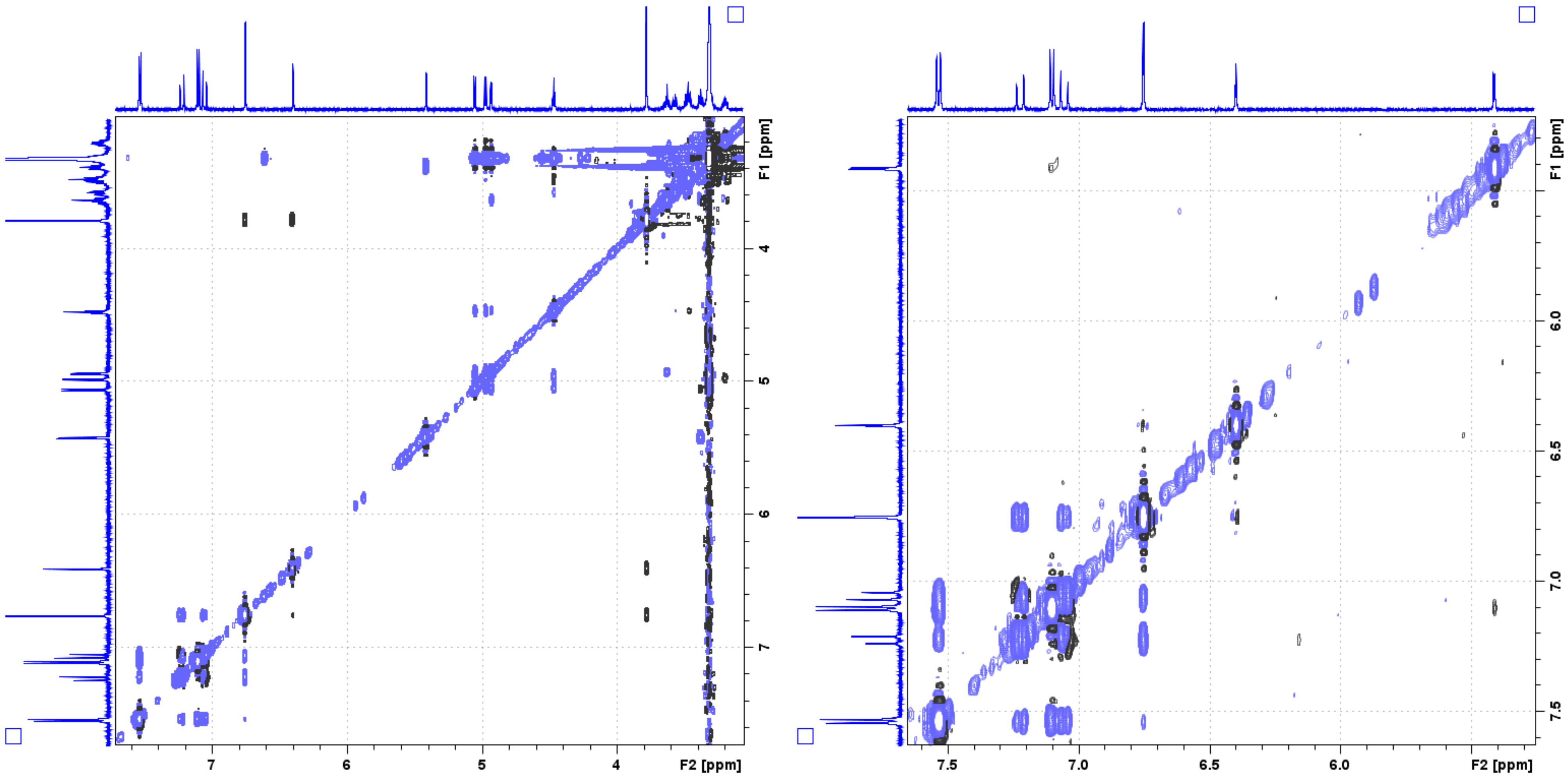

Figure S4.

HRMS and elementary composition calculation for pterostilbene 4′-O-α-D-glucopyranoside.

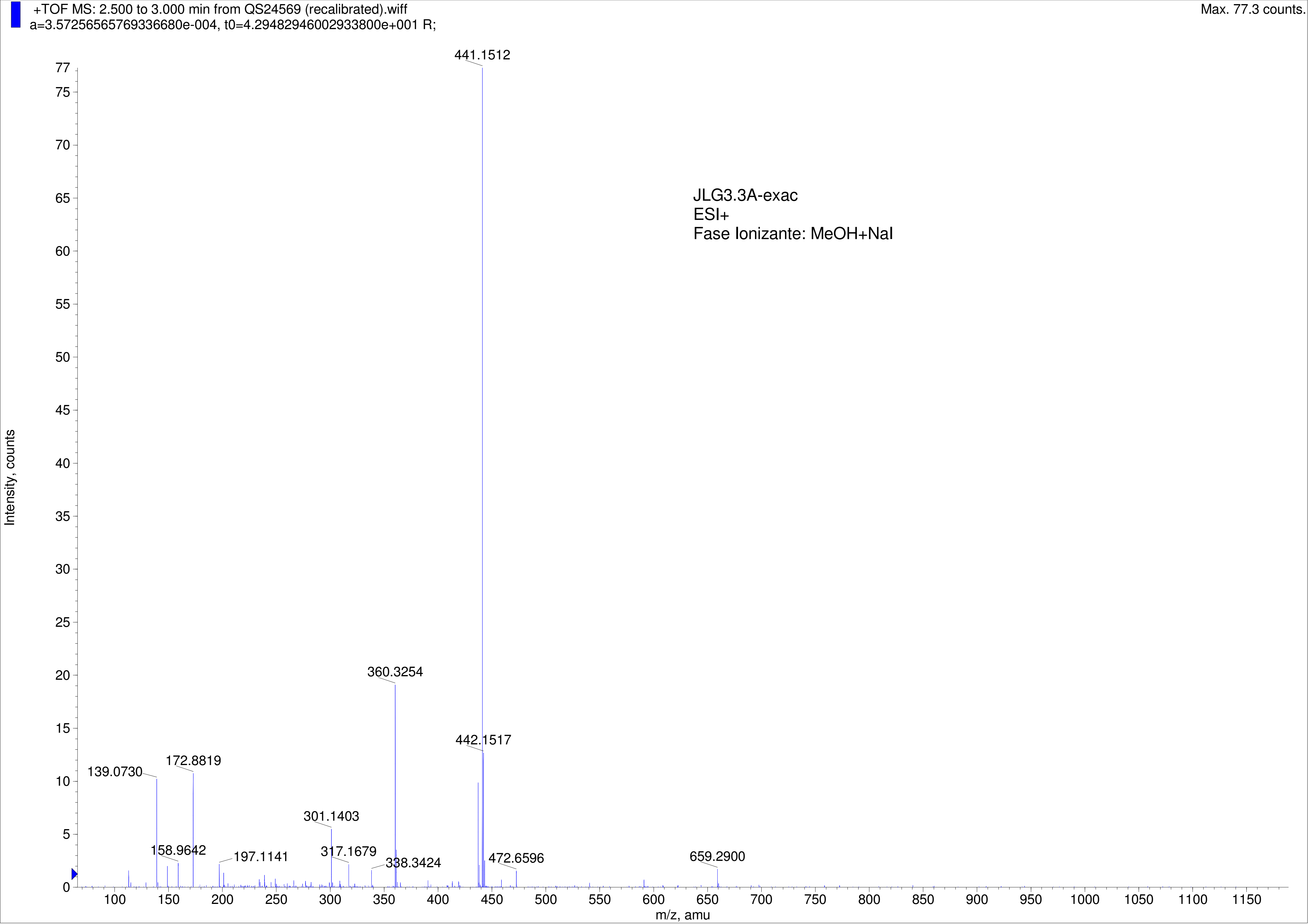

+TOF MS: 2.500 to 3.000 min from QS24569 (recalibrated).wiff
a=3.57256565769336680e-004, t0=4.29482946002933800e+001 R;
Max. 77.3 counts.
JLG3.3A-exac
ESI+
Fase Ionizante: MeOH+NaI
441.1512
360.3254
442.1517
139.0730
172.8819
301.1403
158.9642
197.1141
317.1679
338.3424
472.6596
659.2900
Intensity, counts
m/z, amu

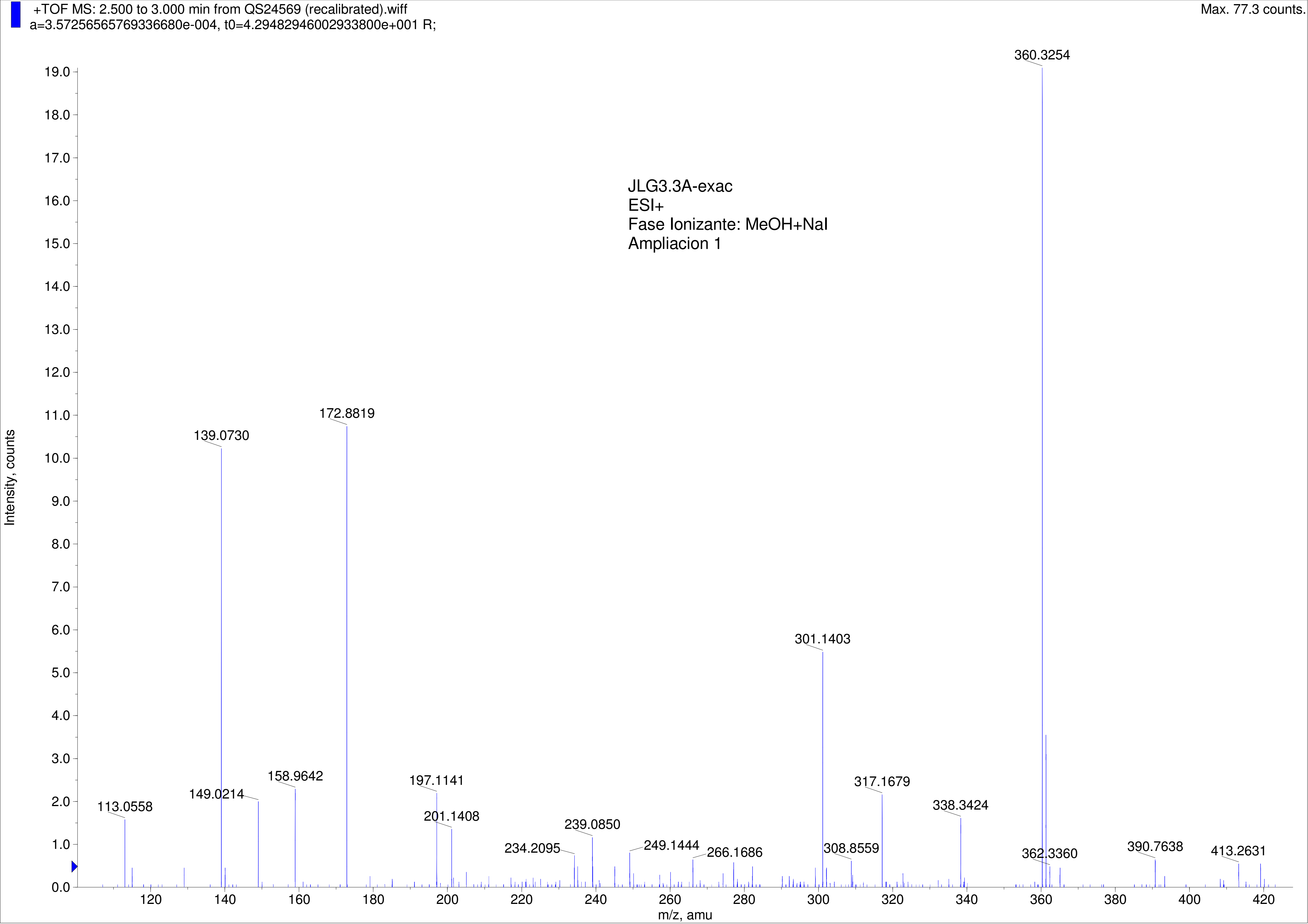

+TOF MS: 2.500 to 3.000 min from QS24569 (recalibrated).wiff
a=3.57256565769336680e-004, t0=4.29482946002933800e+001 R;
Max. 77.3 counts.
JLG3.3A-exac
ESI+
Fase Ionizante: MeOH+NaI
Ampliacion 1
360.3254
172.8819
139.0730
301.1403
158.9642
149.0214
197.1141
317.1679
113.0558
201.1408
338.3424
239.0850
234.2095
249.1444
266.1686
308.8559
362.3360
390.7638
413.2631
Intensity, counts
m/z, amu

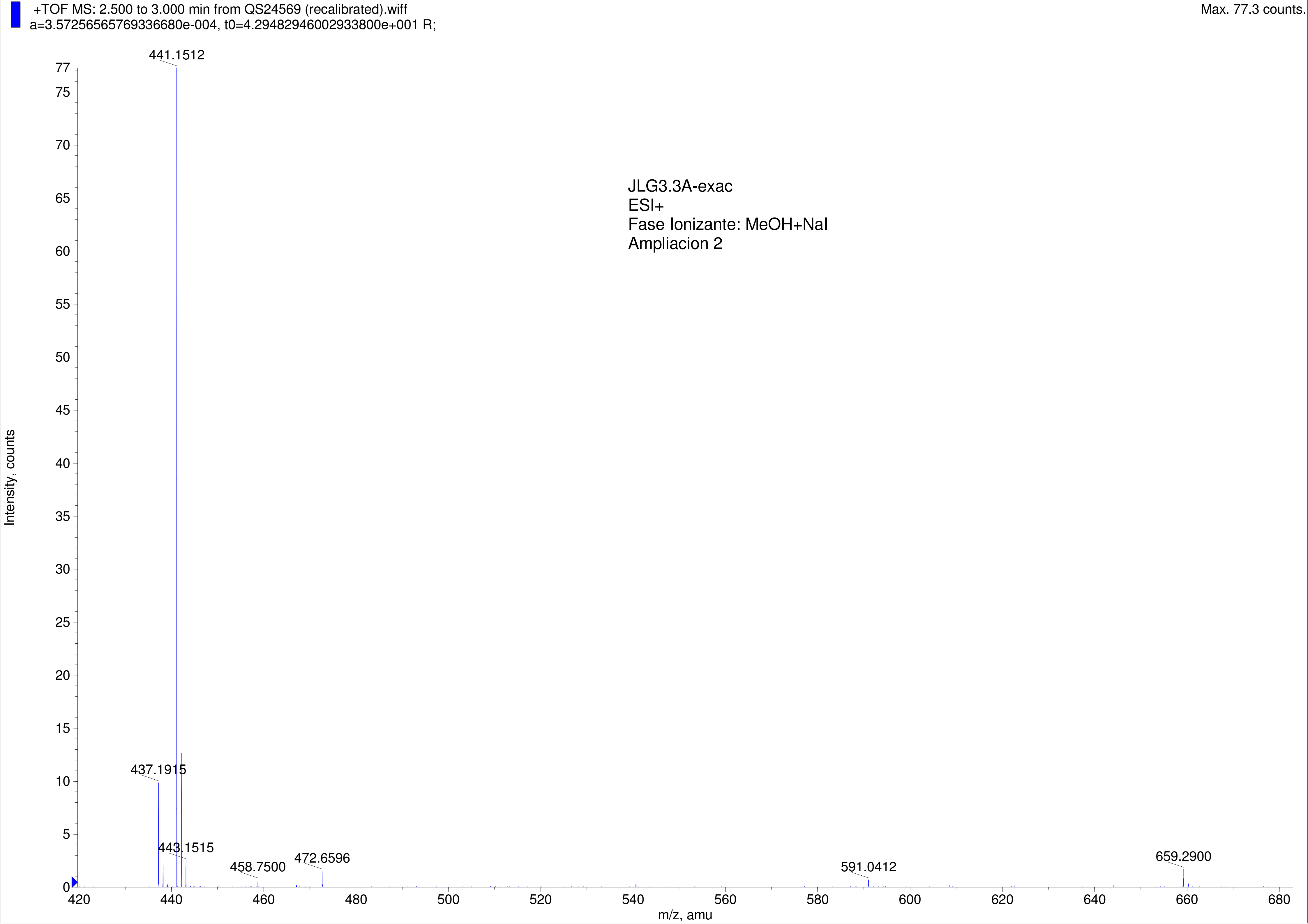
+TOF MS: 2.500 to 3.000 min from QS24569 (recalibrated).wiff
a=3.57256565769336680e-004, t0=4.29482946002933800e+001 R;
Max. 77.3 counts.
JLG3.3A-exac
ESI+
Fase Ionizante: MeOH+NaI
Ampliacion 2
441.1512
437.1915
443.1515
458.7500
472.6596
591.0412
659.2900
Intensity, counts
m/z, amu

Peak List for " +TOF MS: 2.500 to 3.000 min from QS24569 (recalibrated).wiff a=3.57256565769336680e-004, t0=4.29482946002933800e+001 R;"

	Centroid mass (amu)	Centroid intensity	Charges	%Intensity
1	113.0558	1.8280	Undefined	2.0451
2	139.0730	12.1720	Undefined	13.2304
3	149.0214	2.5269	Undefined	2.5876
4	158.9642	2.7634	Undefined	2.9633
5	172.8819	16.4731	Undefined	13.8982
6	197.1141	2.6667	Undefined	2.8381
7	201.1408	1.3548	Undefined	1.7529
8	234.2095	0.6183	Undefined	0.9599
9	235.0948	0.3978	Undefined	0.6260
10	239.0850	1.3871	Undefined	1.5025
11	245.0788	0.6022	Undefined	0.6260
12	249.1444	0.7742	Undefined	1.0434
13	266.1686	0.6989	Undefined	0.8347
14	277.1427	0.7204	Undefined	0.7513
15	282.2085	0.4731	Undefined	0.6260
16	301.1403	8.4785	Undefined	7.0952
17	308.8559	0.5645	Undefined	0.7930
18	317.1679	2.9194	Undefined	2.7963
19	338.3424	2.3226	Undefined	2.0868
20	360.3254	30.5914	1	24.7078
21	361.3284	5.7634	1	4.5910
22	362.3360	0.5430	Undefined	0.6260
23	390.7638	1.0000	Undefined	0.8347
24	413.2631	0.6613	Undefined	0.7095
25	419.1647	0.8172	Undefined	0.7095
26	437.1915	17.1398	1	12.7713
27	438.1961	3.3978	1	2.7129
28	441.1512	140.1828	1	100.0000
29	442.1517	26.4516	1	16.4023
30	443.1515	3.3978	1	3.2554
31	458.7500	1.0161	Undefined	0.9182
32	472.6596	2.2366	Undefined	2.0033
33	591.0412	1.1183	Undefined	0.9182
34	659.2900	2.2688	Undefined	2.2120

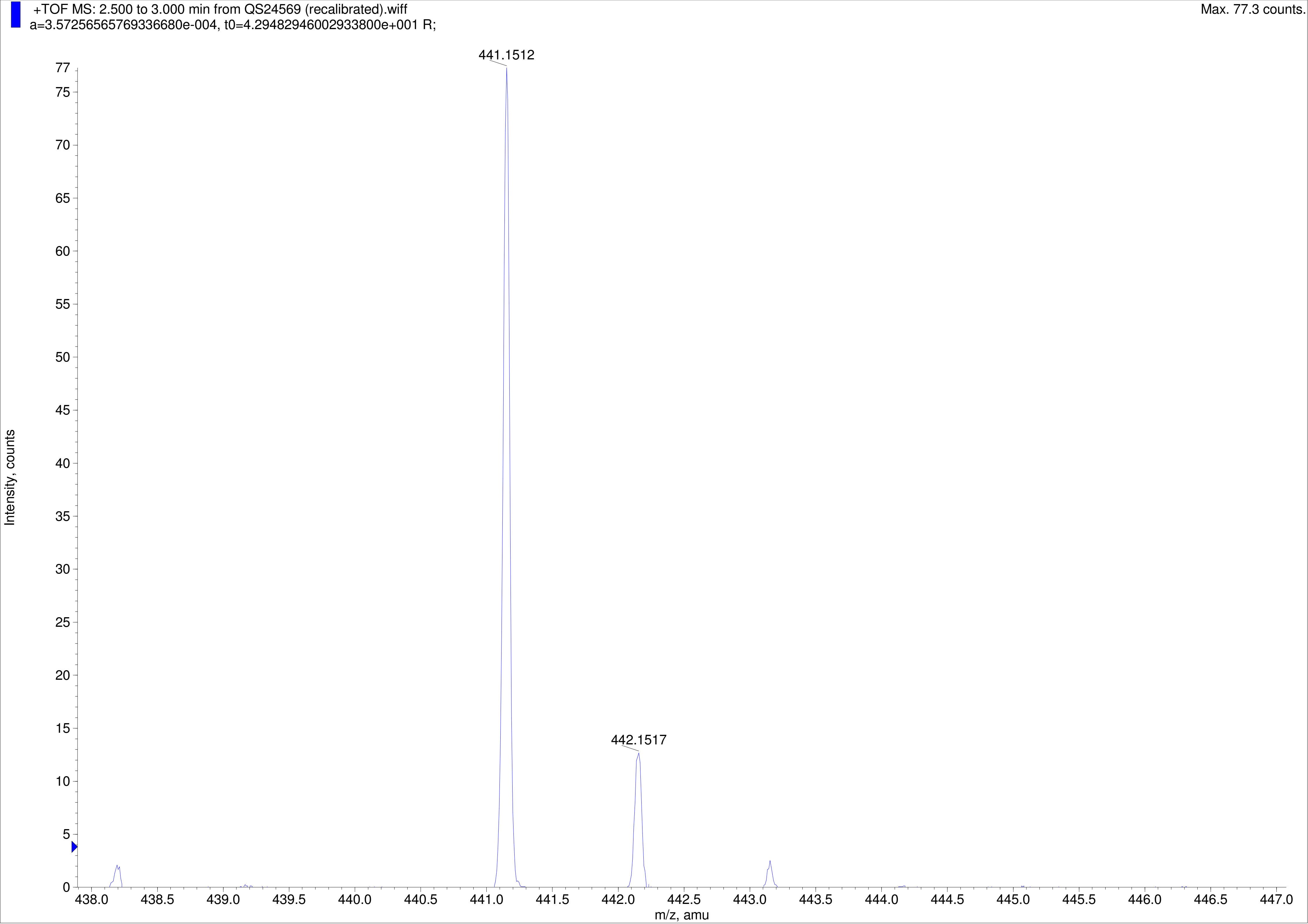
+TOF MS: 2.500 to 3.000 min from QS24569 (recalibrated).wiff
a=3.57256565769336680e-004, t0=4.29482946002933800e+001 R;
Max. 77.3 counts.
441.1512
442.1517
Intensity, counts
m/z, amu

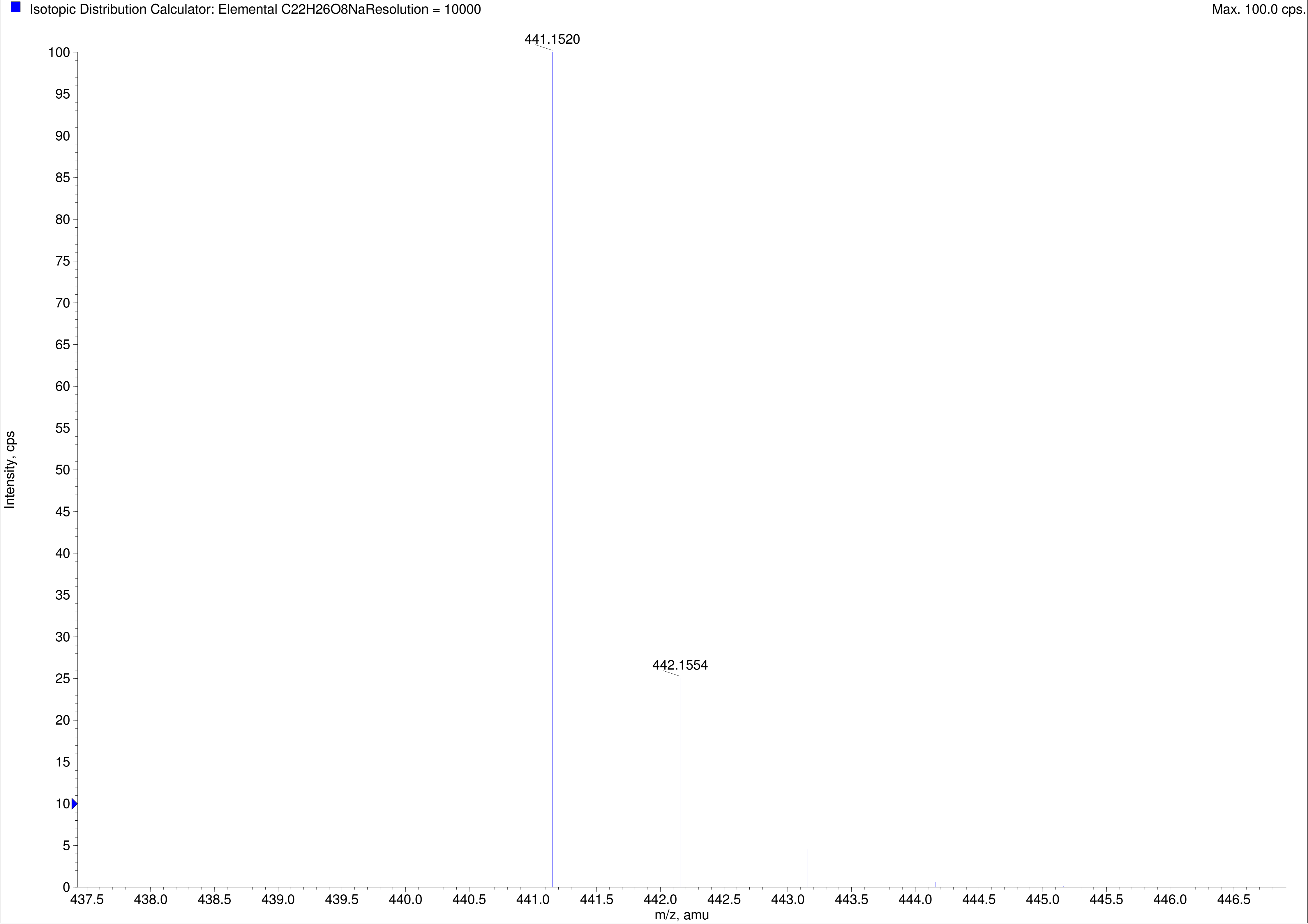
Isotopic Distribution Calculator: Elemental C22H26O8NaResolution = 10000
Max. 100.0 cps.
441.1520
442.1554
Intensity, cps
m/z, amu

Elemental composition calculator

Target m/z: +441.1512 amu
Tolerance: +10.0000 ppm
Result type: Elemental
Max num of results: 20
Min DBE: -2.0000 Max DBE: +100.0000
Electron state: Even
Num of charges: 1
Add water: N/A
Add proton: N/A

	Elements	Min Number	Max Number
1	C	0	100
2	H	0	100
3	O	0	10
4	Na	1	1

	Formula	Calculated m/z (amu)	mDa Error	PPM Error	DBE
1	C22 H26 O8 Na	441.1519	-0.7892	-1.7891	9.5
